# Anti‐Compensatory Saccades Changes After Visuo‐Vestibular Physical Therapy in People With Acute Unilateral Vestibulopathy: A Prospective Observational Study

**DOI:** 10.1002/pri.70274

**Published:** 2026-06-30

**Authors:** Marco Boldreghini, Andrea Canale, Andrea Albera, Simona Minichiello, Claudia Cassandro, Giancarlo Pecorari, Paolo Tasca, Diletta Balta, Marco Caruso, Andrea Cereatti, Marco Tramontano

**Affiliations:** ^1^ Otoneurophysiology and Biomechanics of Human Movement Lab, Department of Surgical Sciences University of Turin Turin Italy; ^2^ ENT Unit Azienda Ospedaliero Universitaria Città della Salute e della Scienza di Torino Torino Italy; ^3^ Department of Electronics and Telecommunications (DET) Politecnico of Turin Torino Italy; ^4^ Translational Rehabilitation Group, Department of Biomedical and Neuromotor Sciences (DIBINEM) Alma Mater Studiorum University of Bologna Bologna Italy; ^5^ Unit of Occupational Medicine IRCCS Azienda Ospedaliero‐Universitaria di Bologna Bologna Italy

## Abstract

**Background and Purpose:**

Acute unilateral vestibulopathy (AUVP) impairs the vestibulo‐ocular reflex (VOR), leading to gaze instability and significant functional disability. Anti‐compensatory saccades (AcS) assessed using the Suppression Head Impulse Paradigm (SHIMP) reflect vestibulo‐saccadic interaction and may represent a marker of central compensation. The aim of the study was to analyze changes in AcS following structured visuo‐vestibular physical therapy (VVPT).

**Methods:**

Ninety‐two patients with AUVP (mean age 59 years, 57 women and 35 men) completed an 8‐week VVPT program (one session per week). Assessments at baseline (T0) and post‐intervention (T1) included the video Head Impulse test (vHIT‐ HIMP and SHIMP) and the Dizziness Handicap Inventory (DHI). The primary outcome was the change in AcS amplitude. Secondary outcomes included changes in VOR gain and DHI score. Paired comparisons were performed using the Wilcoxon signed rank‐test.

**Results:**

AcS amplitude increased significantly after VVPT (median 189.5°–239.0°; *p* < 0.001). VOR gain showed a modest but significant increase (0.37–0.40; *p* < 0.001), remaining within dysfunctional ranges. DHI scores decreased markedly (51–26; *p* < 0.001). No significant association was found between baseline VOR gain and AcS variation (*R* ^2^ = 0.001; *p* = 0.723), suggesting that baseline residual vestibular function did not explain the magnitude of the observed AcS change.

**Discussion:**

A significant increase in AcS amplitude was observed after an 8‐week VVPT program in people with AUVP, despite only limited improvement in VOR gain. These findings suggest that AcS may provide clinically relevant information on vestibulo‐saccadic adaptation during recovery after unilateral vestibular loss and may complement traditional VOR gain and patient‐reported outcome measures. However, given the study design, the observed changes should be interpreted with caution.

## 1 Introduction

Acute Unilateral Vestibulopathy (AUVP), also called vestibular neuritis, is one of the most common and debilitating peripheral vestibular syndromes (Strupp and Brandt 2009). It is characterized by a sudden and asymmetrical loss of peripheral vestibular input, resulting in an alteration of the vestibulo‐ocular reflex (VOR).

This dysfunction causes acute symptoms dominated by vertigo, gaze instability, nausea, and a significant reduction in functional autonomy (Strupp et al. 2020).

From a pathophysiological point of view, VOR deficit compromises the ability to stabilize the image on the retina during rapid head movements, generating retinal slip and requiring the activation of oculomotor compensatory mechanisms (Aw et al. 1996).

Over the last 2 decades, the introduction of the vHIT has enabled objective, quantitative, and reproducible assessment of semicircular canal function and high‐frequency VOR (Weber et al. 2009). The VOR activation paradigm of the vHIT (Head Impulse Paradigm—HIMP) allows the measurement of the angular VOR gain and the identification of compensatory saccades in the presence of a reduction in VOR gain. However, VOR gain alone is not always sufficient to comprehensively describe the functional status of the vestibular system or the adaptive processes that characterize vestibular compensation over time.

In this context, the Suppression Head Impulse Paradigm (SHIMP) has introduced a relevant conceptual change in the assessment of vestibular function (MacDougall et al. 2016). In the Head Impulse Paradigm (HIMP), individuals are instructed to fixate an earth‐fixed visual target so that an intact vestibulo‐ocular reflex generates compensatory eye movements that stabilize gaze during head impulses. In contrast, in the SHIMP paradigm, individuals fixate a head‐fixed visual target that moves synchronously with the head. Under conditions of preserved vestibular function, activation of the VOR drives the eyes away from the target, thereby requiring a subsequent anti‐compensatory saccade (AcS) to re‐establish fixation (Shen et al. 2016). Conversely, in patients with marked semicircular canal impairment, VOR activation is reduced. As a result, the eyes are displaced less from the head‐fixed target during SHIMP, and the subsequent anti‐compensatory saccades may therefore be smaller or absent. However, SHIMP responses are not solely determined by the degree of canal impairment, as they may also vary according to residual vestibular function and individual adaptive strategies. AcS are therefore an indirect but sensitive indicator of vestibulo‐saccadic interaction and residual vestibular function (Casani et al. 2021; Manzari et al. 2022). Previous studies have shown that the amplitude and speed of AcS are related to the state of vestibular compensation and may reflect not only the presence of residual canal input but also the activation of adaptive oculomotor strategies (MacDougall et al. 2016; Manzari et al. 2022). In particular, it has been hypothesized that AcS derives from a combination of two main mechanisms: on the one hand, a VOR‐dependent component, linked to the persistence or partial recovery of canal function; on the other hand, a VOR‐independent component associated with central adaptation and oculomotor reorganization processes (MacDougall et al. 2016; Colagiorgio et al. 2017).

Vestibular physical therapy is currently considered the first‐line treatment to promote functional recovery after AUVP (Giray et al. 2009; Jacobson and Newman 1990; Nola et al. 2010). Vestibular physical therapy principles consisted of adaptation, substitution, and habituation exercises. More recently, increasing attention has been directed toward visuo‐vestibular interaction strategies designed to specifically engage oculomotor mechanisms and stimulate the VOR substitution (Tramontano et al. 2025). However, growing evidence indicates that the clinical and functional improvements observed after vestibular physical therapy cannot be fully explained by changes in passive VOR gain. In particular, Millar and colleagues (Millar et al. 2020) demonstrated that significant gains in subjective outcomes and dynamic visual performance may occur despite the absence of measurable improvements in passive VOR gain, suggesting the involvement of alternative compensatory mechanisms beyond gaze stability recovery. In this context, increasing attention has been directed toward rehabilitation approaches incorporating specific visuo‐vestibular strategies designed to actively stimulate gaze stabilization (Tramontano et al. 2025). These strategies are thought to promote substitution mechanisms based on adaptive saccadic behavior rather than relying primarily on VOR adaptation training. Despite robust evidence supporting the clinical effectiveness of rehabilitation in reducing symptoms and perceived disability, its specific effects on saccadic dynamics, particularly on AcS assessed using the SHIMP, remain insufficiently explored.

To date, few studies have systematically investigated whether structured Visuo‐Vestibular interventions within Physical Therapy (VVPT) are capable of modifying AcS, and whether such modifications reflect vestibular reflex recovery or predominantly central, oculomotor adaptive processes. Importantly, a previous study (Manzari and Tramontano 2020) demonstrated that even a minimal improvement in VOR gain during SHIMP testing may represent a trigger for the reappearance of AcS, indicating a reactivation of vestibulo‐saccadic interaction despite persistently reduced VOR gain. Against this background, the present prospective observational study investigated pre–post changes in AcS amplitude after an 8‐week structured VVPT program in individuals with AUVP. The primary hypothesis was that AcS amplitude would change over the observation period. Secondary objectives were to assess concurrent changes in VOR gain and perceived disability, and to explore the relationship between baseline residual vestibular function and changes in AcS amplitude.

By integrating instrumental vestibular measures with clinical outcomes, this study seeks to clarify the specific role of substitutive oculomotor strategies within VVPT. In this framework, AcS may represent a sensitive functional marker of vestibulo‐saccadic adaptation during recovery and may complement traditional vestibular outcome measures in the assessment of patients undergoing VVPT (Manzari et al. 2022).

## 2 Methods

### 2.1 Study Design

This prospective observational study used a pre–post intervention design to investigate changes in AcS following a structured VVPT program in individuals with AUVP. The study was conducted and reported in accordance with the Strengthening the Reporting of Observational Studies in Epidemiology (STROBE) guidelines and approved by the Territorial Ethics Committee AOU Città della Salute e della Scienza of Turin with Prot. n. 1300 27/03/2025. Principles of the Declaration of Helsinki and current national legislation were followed. Written informed consent was obtained from all participants for the publication of results. All participants underwent a baseline assessment (T0) and a post‐intervention assessment (T1) 8 weeks later. Accordingly, the study was designed to describe changes observed over time after VVPT rather than to establish the specific causal efficacy of the intervention. Spontaneous vestibular compensation and natural recovery after AUVP were therefore considered as potential contributors to the observed changes.

### 2.2 Participants

Participants were consecutively recruited from the Vestibology Unit of Molinette Hospital (Turin, Italy). Individuals were eligible for inclusion if they met the following criteria: (i) diagnosis of acute unilateral vestibulopathy based on clinical presentation and instrumental vestibular testing; (ii) sub‐acute phase (> 72 h) at the time of enrollment; (iii) documented unilateral peripheral vestibular deficit at vHIT; (iv) ability to understand and perform the proposed rehabilitation exercises; and (v) willingness to complete the full intervention protocol.

Exclusion criteria included the presence of central neurological disorders, non‐vestibular oculomotor disorders, significant cognitive impairment, musculoskeletal conditions limiting participation in rehabilitation, and previous participation in vestibular rehabilitation programs within the months preceding enrollment.

### 2.3 Instrumental Assessment Protocol

Instrumental vestibular assessment was performed using vHIT employing HIMP and SHIMP paradigms for integrated analysis of the VOR and saccadic strategies (Weber et al. 2009; MacDougall et al. 2016). The tests were conducted by experienced operators under standardized conditions following international recommendations.

The ICS Impulse system (software Otosuite Vestibular) was used to measure head and eye velocity during both tests.

In the HIMP paradigm, individuals were asked to fixate on a static visual target positioned in front of them, while rapid and unpredictable head impulses were applied in the horizontal plane. VOR gain was calculated as the ratio of eye velocity to head velocity, considering only technically valid impulses. The VOR gain and AcS parameters were extracted using proprietary algorithms.

In the SHIMP paradigm, the visual target moved in unison with the patient's head, inducing a vestibular response that required the execution of an AcS to re‐establish fixation. AcS amplitude was extracted from SHIMP trials on the affected side and expressed in degrees (°). For each participant and time point, the mean AcS amplitude across valid impulses was used for statistical analysis. AcS were identified using the proprietary algorithm of the ICS Impulse/Otosuite Vestibular software and visually checked by an experienced examiner to exclude artifacts or incorrectly detected responses. All T0 and T1 assessments were performed by the same experienced operator using the same standardized acquisition and quality‐control procedures (Manzari et al. 2022; Colagiorgio et al. 2017).

### 2.4 Clinical Evaluation

Perceived disability related to balance disorders was assessed using the validated [Italian‐language] version of the Dizziness Handicap Inventory (DHI), a tool that investigates the impact of vestibular symptoms on the functional, emotional, and physical dimensions of daily life (Jacobson and Newman 1990; Nola et al. 2010). The questionnaire was administered in both assessment phases (T0 and T1), allowing for an analysis of the subjective evolution of symptoms.

### 2.5 Interventions

All participants performed 30‐min sessions once weekly for 8 weeks to improve gaze stability through sensorimotor substitution. The intervention was based on updated principles of vestibular physical therapy, emphasizing gaze stability, sensory substitution, and cognitive–motor integration during dynamic tasks (Tramontano et al. 2025). Dynamic‐unpredictable saccadic training was designed to promote rapid gaze relocation under conditions of visual and motor unpredictability. Exercises included rapid shifts of gaze toward visual targets presented at variable timing and spatial locations, and SHIMP‐like substitution tasks in which patients were required to follow a visual target during unpredictable head movements. When appropriate, laser‐based exercises were used to provide an external visual target during head and body movements, consistent with previously described visuo‐vestibular substitution strategies (Tramontano et al. 2025). Cognitive–motor dual‐task exercises were included to reproduce more ecological conditions of daily life. These tasks combined gaze stabilization or gaze‐shift exercises with concurrent motor or cognitive demands, such as standing balance tasks, walking, target reaching, and counting. The rationale was to challenge gaze and postural control under conditions requiring divided attention and sensorimotor integration.

The type and general structure of the exercises were standardized across participants, whereas intensity and progression were individualized according to symptom tolerance, postural control, and correct execution of the required oculomotor strategy. Progression was based on increasing exercise load and complexity, including head movement velocity, range of motion, exercise duration, visual target unpredictability, postural challenge, and the introduction of dual‐task conditions. Exercises were simplified or temporarily reduced when symptoms were excessive, prolonged, or interfered with correct task execution. Baseline instrumental vestibular parameters, including VOR gain, were not used to prescribe or modify the rehabilitation program, in order to avoid biasing the exploratory analysis of the relationship between residual vestibular function and AcS change. Participants were also instructed to perform a home exercise program based on the same principles practiced during supervised sessions. Home exercises included gaze stabilization and saccadic substitution tasks during daily activities, such as standing, walking, turning the head, or moving in visually complex environments, according to individual tolerance. Exercise execution and adherence were reviewed at each supervised session through patient reports, and the program was adapted when needed.

### 2.6 Outcome

The primary outcome of the study was the pre‐post change in AcS amplitude assessed using SHIMP on the affected side between T0 and T1.

Secondary outcomes included the change in VOR gain measured using the HIMP paradigm and changes in the DHI score between T0 and T1. The possible relationship between baseline residual vestibular function, expressed as affected‐side VOR gain, and the observed change in AcS amplitude was explored as a secondary exploratory analysis.

### 2.7 Statistical Analysis

All primary and secondary analyses were conducted within a paired‐samples statistical framework, reflecting the repeated assessment of outcomes before and after the intervention. Accordingly, all inferential procedures were chosen to account for the dependency between observations collected at different time points.

The normality of the data was preliminarily assessed using the Shapiro–Wilk test, applied to the residuals of each paired comparison. This assessment indicated a deviation from normality for all the outcome variables. Considering these findings, comparisons between T0 and T1 were performed using the Wilcoxon signed‐rank test for paired data.

For Wilcoxon signed‐rank tests, effect size was quantified using rank‐biserial correlation. Rank‐biserial correlation was calculated from the signed ranks as the difference between the sums of positive and negative ranks divided by their total sum. The sign of the rank‐biserial correlation reflects the direction of the paired difference according to the order of the comparison, whereas the absolute value reflects the magnitude of the effect. Therefore, negative values indicate an increase from T0 to T1 when the comparison is expressed as T0 minus T1, while positive values indicate a decrease from T0 to T1.

Given the limited number of predefined outcomes and the exploratory nature of secondary analyses, no formal correction for multiple comparisons was applied. The results of secondary and correlation analyses should therefore be interpreted as exploratory. Linear regression was used to assess whether baseline affected‐side VOR gain predicted the change in AcS amplitude, calculated as the difference between AcS amplitude at T1 and T0.

The level of statistical significance was set at *p* < 0.001. The analyses were performed using JASP, a free open‐source statistical software built on *R* developed by researchers at the University of Amsterdam (JASP Team 2025).

## 3 Results

Ninety‐two patients with AUVP were enrolled (57 females; mean age 59.1 ± 10.4 years). Forty‐eight participants were affected on the left side and 44 on the right. Enrollment occurred during the post‐acute phase, with a mean interval of 61 ± 17 days from symptom onset. At baseline (T0), VOR gain on the affected side was reduced (0.37 ± 0.23), consistent with vestibular deficit. The sample characteristics are summarized in Table 1.

**TABLE 1:** Demographic and clinical characteristics of the sample.

AUVP	48 L; 44 R
Sex	57 F; 35 M
Age (mean ± SD years)	59.1 ± 10.4
Time from symptom onset (mean ± SD days)	61 ± 17
Ipsilateral horizontal VOR gain (mean and SD)	0.37 ± 0.23

Abbreviations: AUVP = acute unilateral vestibulopathy, F = Female, L = Left, M = Male, R = Right, SD = Standard deviation.

Results of descriptive statistics for the analyzed dataset are reported in Table 2.

**TABLE 2:** Descriptive statistics.

	AcS_amp_ T0	AcS_amp_ T1	VOR T0	VOR T1	DHI T0	DHI T1
Median	189.5	239.0	0.37	0.40	51.0	26.0
IQR	47.3	60.0	0.23	0.17	19.5	15.0
Minimum	0.0	102.0	0.04	0.11	6.0	2.0
Maximum	363.0	420.0	0.79	0.87	92.0	58.0

Abbreviations: AcS_amp_ = AcS anti‐compensatory saccade amplitude, DHI = Dizziness Handicap Inventory, IQR = inter‐quartile range, T0 = baseline, T1 = post intervention, VOR = vestibulo‐ocular reflex gain.

Data analysis revealed statistically significant pre–post changes in all predefined outcomes over the 8‐week observation period. AcS amplitude showed a notable change between T0 and T1 (Wilcoxon *W* = 82.5, *z* = −8.008, *p* < 0.001), with a very large effect size (*r* = −0.961). Similarly, VOR gain also showed a statistically significant variation (*W* = 822.5, *z* = −3.946, *p* < 0.001), suggesting a change that is present but more limited, with a moderate effect size (*r* = −0.505). Finally, DHI scores demonstrated a significant reduction between T0 and T1 (*W* = 4002.0, *z* = 8.181, *p* < 0.001), with a very large effect size (*r* = 0.999), indicating a significant reduction in perceived disability. All variables were assessed by T0 and T1 measurements, allowing the quantification of within‐individual change over time. These results are reported in Table 3.

**TABLE 3:** Results of the Wilcoxon signed‐rank tests.

	*W*	*z*	*p*	$\bar{x}$	*r*
AcS amplitude T0–AcS amplitude T1	82.5	−8.0	< 0.001	−50.5	−0.961
VOR T0–VOR T1	822.5	−3.9	< 0.001	−0.03	−0.505
DHI T0–DHI T1	4002.0	8.2	< 0.001	22.0	0.999

*Note:* Results of the Wilcoxon signed‐rank tests for pre–post comparisons between baseline assessment (T0) and post‐intervention assessment (T1). The sign of the median paired difference and of *r* reflects the direction of the paired comparison, whereas the absolute value of *r* reflects effect magnitude.

Abbreviations: AcS amplitude = anti‐compensatory saccade amplitude assessed using the Suppression Head Impulse Paradigm, DHI score = Dizziness Handicap Inventory score, Median paired difference = median paired difference calculated as T0 minus T1, *p* = *p*‐value, *r* = rank‐biserial correlation effect size, VOR gain = vestibulo‐ocular reflex gain assessed using the Head Impulse Paradigm, *W* = Wilcoxon signed‐rank test statistic, *z* = standardized Wilcoxon test statistic.

**FIGURE 1:**
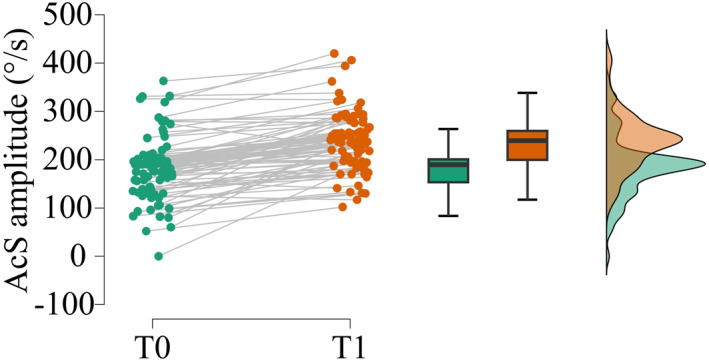
AcS amplitude. Anti‐compensatory saccade (AcS) amplitude at baseline (T0) and post‐intervention assessment (T1) in patients with acute unilateral vestibulopathy. Values refer to SHIMP responses on the affected side. Individual paired observations are overlaid and connected by lines to illustrate within‐participant changes over time. A comparison was done between T0 and T1 using the Wilcoxon signed‐rank test.

The main outcome of the study, represented by the amplitude of AcS assessed using the SHIMP paradigm, showed a marked and systematic change after the VVPT program (as reported in Figure 1).

Analysis using the Wilcoxon test for paired data confirmed the presence of a statistically significant difference between AcS amplitude values observed at T0 and T1 (*p* < 0.001), accompanied by a large effect size (rank‐biserial correlation *r* = −0.961). The median AcS amplitude value increased from 189.5° at time T0 to 239.0° at time T1.

**FIGURE 2:**
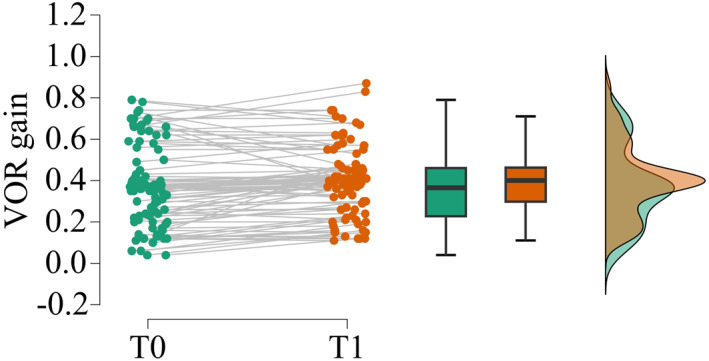
VOR gain. Vestibulo‐ocular reflex (VOR) gain at baseline (T0) and post‐intervention assessment (T1) in patients with acute unilateral vestibulopathy. Values refer to affected‐side horizontal canal responses measured using the HIMP paradigm. Individual paired observations are overlaid and connected by lines to illustrate within‐participant changes over time. The comparison between T0 and T1 was performed using the Wilcoxon signed‐rank test.

This finding suggests a large and consistent increase in AcS amplitude over the observation period. A statistically significant increase in VOR gain measured using the HIMP paradigm was observed *p* < 0.001 (Figure 2). The median VOR gain value increased from 0.37 at time T0 to 0.40 at time T1.

Observed differences were statistically significant (*p* < 0.001) with a large effect size (*r* = −0.505). However, the post‐intervention values (< 0.90) remain compatible with a condition of residual semicircular canal impairment, indicating that the improvement in VOR, although present, does not reach complete physiological levels.

**FIGURE 3:**
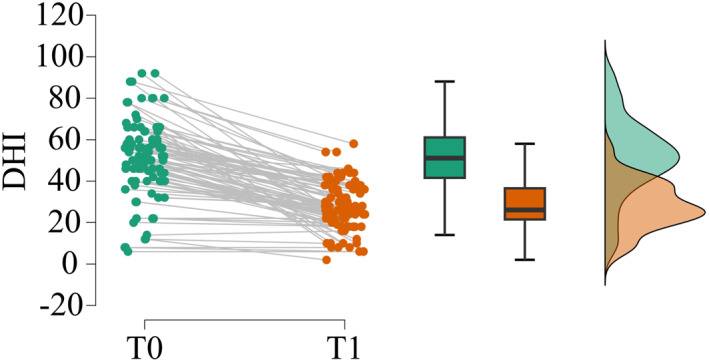
DHI. Dizziness Handicap Inventory (DHI) scores at baseline (T0) and post‐intervention assessment (T1). Individual paired observations are overlaid and connected by lines to illustrate within‐participant changes over time. Lower DHI scores indicate lower perceived disability. A comparison between T0 and T1 was done using the Wilcoxon signed‐rank test.

Clinical assessment using the Dizziness Handicap Inventory showed a statistically significant reduction in perceived disability associated with balance disorders (Figure 3). The median DHI score decreased from 51 points at time T0 to 26 points at time T1.

Again, the statistical comparison showed extremely high significance (*p* < 0.001), with a very large effect size (*r* = 0.999).

### 3.1 Correlation and Regression Analysis

**FIGURE 4:**
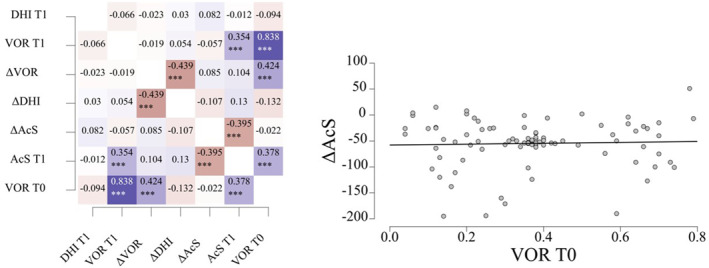
Correlation/regression analyses. The map on the left illustrates the strength and direction of associations among the study variables (AcSamp = anti‐compensatory saccade amplitude, VOR = vestibulo‐oculomotor reflex gain, DHI = Dizziness Handicap Inventory score) and their variations over the rehabilitation protocol (ΔAcS = change in anti‐compensatory saccade amplitude, ΔVOR = change in vestibulo‐oculomotor reflex gain, ΔDHI = change in Dizziness Handicap Inventory score). Color intensity reflects the magnitude of the correlation coefficients (blue: maximally positive correlation; red: maximally negative correlation). Three asterisks (***) indicate statistically significant correlations (*p* < 0.001). The graph on the right shows the relationship between baseline vestibulo‐oculomotor reflex gain (VOR T0) and the change in saccadic movement amplitude (ΔAcS = change in anti‐compensatory saccade amplitude). Each point represents an individual participant, and the solid line indicates the fitted linear regression.

To explore the relationship between the different outcomes, correlation analyses were performed (Figure 4). No significant correlation emerged between the variation in AcS amplitude (ΔAcS) and changes in VOR gain (0.085), nor between ΔAcS and the reduction in the DHI score (−0.107). Instead, a significant moderate correlation was observed between the amplitude of AcS and DHI score after rehabilitation (0.354) and between the reduction of DHI score and the increase in VOR gain (−0.439).

In particular, the exploratory linear regression analysis showed that baseline VOR gain was not a significant predictor of the observed change in AcS amplitude over the rehabilitation period (*R* ^2^ = 0.001; *p* = 0.723).

## 4 Discussion

This study identified pre–post changes in AcS amplitude in individuals with AUVP assessed before and after an 8‐week VVPT program. The main finding was a marked and consistent increase in AcS amplitude over the observation period, accompanied by a significant reduction in perceived disability and only modest changes in VOR gain. Given the uncontrolled observational design, these findings should be interpreted as changes observed after VVPT rather than as definitive evidence of a specific causal treatment effect. Nevertheless, the dissociation between AcS changes and VOR gain recovery represents a clinically relevant observation. AcS assessed using the SHIMP reflects the interaction between residual vestibular input and central oculomotor control mechanisms rather than a mere by‐product of VOR function. In line with previous studies, AcS amplitude and occurrence appear to capture adaptive processes that emerge during vestibular compensation and are not fully explained by changes in VOR gain alone (MacDougall et al. 2016; Manzari et al. 2022; Colagiorgio et al. 2017). Although a statistically significant increase in VOR gain was detected after VVPT, post‐intervention values remained compatible with a condition of residual semicircular canal deficit. This pattern is consistent with previous evidence showing that VOR recovery after acute unilateral vestibulopathy is often partial and variable, even in the presence of substantial clinical improvement (Manzari and Tramontano 2020). The natural history of AUVP should be carefully considered when interpreting these findings. Spontaneous vestibular compensation commonly occurs after unilateral vestibular loss and may involve partial recovery of peripheral function, central recalibration of vestibular tone, visual and proprioceptive substitution, and changes in oculomotor strategies. Therefore, part of the observed improvement in AcS amplitude, VOR gain, and DHI score may reflect spontaneous recovery rather than the specific effect of VVPT. The absence of a non‐treated or alternative‐treatment control group prevents separation of rehabilitation‐associated changes from the expected course of recovery after AUVP.

Crucially, growing evidence indicates that functional recovery following vestibular rehabilitation is not necessarily mediated by normalization of passive VOR gain. Millar and colleagues demonstrated that improvements in dynamic visual performance and patient‐reported outcomes may occur despite unchanged passive VOR gain, highlighting the contribution of alternative compensatory mechanisms beyond reflex restoration (Millar et al. 2020). The present findings are consistent with this concept as pronounced changes in saccadic behavior were observed despite only modest VOR gain improvement. This supports the hypothesis that adaptive oculomotor strategies may contribute to functional recovery.

An important conceptual framework for interpreting the present results comes from previous work by Manzari and Tramontano (Manzari and Tramontano 2020), who showed that even a minimal (“tiny”) improvement in VOR gain during SHIMP testing may act as a trigger for the reappearance of AcS, despite persistently pathological VOR values. In this model, a small residual vestibular drive is sufficient to reactivate vestibulo‐saccadic interaction, allowing the oculomotor system to generate effective anti‐compensatory responses.

Our data are consistent with this interpretation; however, they do not directly prove this mechanism. While a slight increase in VOR gain was observed, baseline VOR values did not explain the magnitude of AcS changes over the observation period. This finding may suggest that factors other than baseline residual VOR gain contribute to AcS modulation. However, it should not be interpreted as definitive evidence that VVPT primarily acts through VOR‐independent central mechanisms.

The substantial reduction in DHI scores observed after the rehabilitation period suggests a clinically relevant reduction in perceived disability. However, DHI is a subjective patient‐reported outcome and may be influenced by several factors, including symptom adaptation, behavioral changes, expectations, emotional status, and natural recovery. Therefore, DHI improvement should be interpreted as complementary to instrumental vestibular measures rather than as direct evidence of a specific physiological mechanism. The dissociation between instrumental measures and subjective outcomes further supports the concept that vestibular compensation is a multidimensional process in which reflex recovery, oculomotor adaptation, and perceptual recalibration may follow partially independent trajectories (Jacobson and Newman 1990; Nola et al. 2010).

In this context, VVPT may be interpreted not merely as a strategy aimed at restoring gaze stability through VOR adaptation but also as a rehabilitation approach that may support broader sensorimotor reorganization. Exercises involving target unpredictability, rapid gaze relocation during head movement, and cognitive‐motor dual‐tasking likely challenge and reinforce adaptive oculomotor control, contributing to improved functional autonomy even in the presence of residual vestibular deficits.

The present findings reinforce the value of the SHIMP paradigm as a sensitive tool for capturing adaptive changes in vestibulo‐saccadic behavior that are not reflected by VOR gain alone. AcS may therefore represent a meaningful functional marker of vestibulo‐saccadic compensation and may complement traditional vestibular outcome measures in patients undergoing VVPT. However, further controlled studies are required before AcS can be considered as a specific biomarker of treatment response. A further mechanistic interpretation may be framed within the recent model of vestibular encoding proposed by Manzari, which emphasizes the role of high‐dynamic vestibular signals in responses to brief and rapid head impulses (Manzari 2026). According to this model, SHIMP testing may preferentially engage fast vestibulo‐saccadic processing as it relies on high‐acceleration head impulses and requires rapid gaze relocation. From this perspective, the observed AcS modulation may be compatible with adaptive changes in fast vestibulo‐oculomotor control, even when VOR gain remains impaired. However, the present study did not directly assess peripheral afferent dynamics, jerk‐sensitive encoding, or central vestibular pathway activity. Therefore, this interpretation should be considered a physiological hypothesis derived from previous literature rather than a mechanism directly demonstrated by the present data. From a rehabilitation perspective, these findings support the potential relevance of visuo‐vestibular strategies targeting adaptive gaze control in patients with unilateral vestibulopathy. Rather than focusing exclusively on VOR gain recovery, rehabilitation may also aim to enhance compensatory gaze strategies capable of supporting function in the presence of persistent vestibular impairment.

This study has several limitations. First, the absence of a control group prevents a definitive distinction between VVPT‐associated changes and spontaneous recovery after AUVP. Spontaneous vestibular compensation may have contributed substantially to the observed improvements in AcS amplitude, VOR gain, and DHI score. Second, although the rehabilitation protocol was structured and delivered within routine clinical practice, exercise intensity and progression were individualized, which may introduce variability in treatment exposure. Third, adherence to the home exercise program was monitored clinically but not quantified using objective measures. Fourth, AcS extraction relied on the proprietary software algorithm, although traces were visually checked for quality control. Fifth, the correlation and regression analyses were exploratory and should not be interpreted as definitive evidence of VOR‐independent central adaptation. Finally, DHI is a subjective outcome measure and may be influenced by factors not directly related to vestibular physiology. Future randomized controlled studies with untreated or alternative‐treatment comparison groups are needed to clarify the specific contribution of VVPT to AcS modulation and to define the temporal dynamics of vestibulo‐saccadic adaptation after AUVP.

This prospective observational study showed a significant increase in AcS amplitude after an 8‐week VVPT program in individuals with AUVP. This change was accompanied by a marked reduction in perceived disability and only a modest improvement in VOR gain, which remained within dysfunctional ranges. Baseline VOR gain did not predict the magnitude of AcS change in exploratory regression analysis. However, because of the uncontrolled pre–post design, the observed changes cannot be attributed exclusively to VVPT, and spontaneous vestibular compensation may have contributed to the results. Controlled studies are needed to determine whether AcS can serve as a specific biomarker of rehabilitation response.

## 5 Implications for Physiotherapy Practice

These findings suggest that AcS may provide clinically relevant information on vestibulo‐saccadic adaptation during recovery after unilateral vestibular loss and may complement traditional VOR gain measures and patient‐reported outcome measures. From a physiotherapy perspective, the facilitation of AcS may represent a useful therapeutic target within visuo‐vestibular physiotherapy.

Exercises involving rapid gaze relocation, dynamic and unpredictable saccadic tasks, active head movements, and cognitive–motor dual‐task conditions may help promote adaptive gaze strategies and support vestibular compensation in people with vestibular dysfunction.

## Funding

The authors have nothing to report.

## Ethics Statement

The study was approved by the Territorial Ethics Committee AOU Turin (Prot. n. 1300 27/03/2025).

## Consent

The study was conducted in accordance with the principles of the Declaration of Helsinki and current national legislation. Written informed consent was obtained from all participants for the publication of results.

## Conflicts of Interest

The authors declare no conflicts of interest.

## Data Availability

Data are available upon request to the corresponding author.
